# How *Escherichia coli* Circumvent Complement-Mediated Killing

**DOI:** 10.3389/fimmu.2017.00452

**Published:** 2017-04-20

**Authors:** Afonso G. Abreu, Angela S. Barbosa

**Affiliations:** ^1^Programa de Pós-Graduação em Biologia Parasitária, CEUMA University, São Luís, Brazil; ^2^Programa de Pós-Graduação em Ciências da Saúde, Federal University of Maranhão, São Luís, Brazil; ^3^Laboratory of Bacteriology, Butantan Institute, São Paulo, Brazil

**Keywords:** *Escherichia coli*, complement system, immune evasion, capsule, proteases, Factor H, C4b-binding protein

## Abstract

Complement is a crucial arm of the innate immune response against invading bacterial pathogens, and one of its main functions is to recognize and destroy target cells. Similar to other pathogens, *Escherichia coli* has evolved mechanisms to overcome complement activation. It is well known that capsular polysaccharide may confer resistance to complement-mediated killing and phagocytosis, being one of the strategies adopted by this bacterium to survive in serum. In addition, proteases produced by *E. coli* have been shown to downregulate the complement system. Pic, an autotransporter secreted by different pathogens in the Enterobacteriaceae family, is able to cleave C2, C3/C3b, and C4/C4b and works synergistically with human Factor I and Factor H (FH), thereby promoting inactivation of C3b. Extracellular serine protease P, a serine protease of enterohemorrhagic *E. coli* (EHEC), downregulates complement activation by cleaving C3/C3b and C5. StcE, a metalloprotease secreted by EHEC, inhibits the classical complement-mediated cell lysis by potentiating the action of C1 inhibitor, and the periplasmic protease Prc contributes to *E. coli* complement evasion by interfering with the classical pathway activation and by preventing membrane attack complex deposition. Finally, it has been described that *E. coli* proteins interact with negative complement regulators to modulate complement activation. The functional consequences resulting from the interaction of outer membrane protein A, new lipoprotein I, outer membrane protein W, and Stx2 with proteins of the FH family and C4b-binding protein (C4BP) are discussed in detail. In brief, in this review, we focused on the different mechanisms used by pathogenic *E. coli* to circumvent complement attack, allowing these bacteria to promote a successful infection.

## Introduction

*Escherichia coli* is one of the most widely studied bacterial species. The gastrointestinal tract of newborn infants is colonized shortly after birth with this bacterium, although it rarely causes disease in healthy individuals. As part of the human microbiota, *E. coli* has established a good relationship with its hosts, coexisting pacifically with mutual profit for both the microorganism and the host. However, highly adapted *E. coli* clones have acquired a repertoire of virulence factors that enable colonization and survival within the host, eventually causing severe disease (1). Diverse *E. coli* pathotypes are able to cause epithelial barrier disruption leading to bacterial invasion and migration into the urinary tract. Some of them can eventually reach the bloodstream and spread to host tissues, causing bacteremia and sepsis. Serum resistance is a key attribute of these pathotypes, and their capacity to circumvent the complement system, our first line of defense against invading pathogens, ensures a successful infection process.

Upon infection, the complement cascade can be activated on the surface of a pathogen through three distinct pathways: the alternative, the classical, and the lectin pathways. All of them converge on a common terminal pathway leading to the formation of the membrane attack complex (MAC), causing cell lysis and death. Other effector functions elicited by complement activation include opsonization, recruitment of inflammatory cells, and release of inflammatory mediators [reviewed in Ref. (2)]. To avoid undesired effects on self tissue, complement activation is tightly regulated by a set of soluble and membrane-bound regulators. Factor H (FH), Factor H-like protein-1 (FHL-1), C4b-binding protein (C4BP), decay-accelerating factor, membrane cofactor protein, and complement receptor 1 are regulators of complement activation that share structural similarities, and display decay acceleration of C3 convertase complexes and/or cofactor functional activity by promoting C3b degradation mediated by Factor I (FI). The membrane-anchored CD59 regulator impairs MAC formation, and C1 inhibitor (C1-INH) is a protease inhibitor that inactivates C1r and C1s in the C1 complex, and also mannose-binding lectin (MBL)-associated serine proteases in MBL complexes, thus interfering with both the classical and the lectin pathways. As a consequence, cleavage of C4 and C2 by C1 and MBL is impaired.

Bacteria and their hosts have been coevolving for millions of years, and it is not surprising that many human pathogens have developed diverse strategies to counteract complement system activation in order to successfully colonize the target organism. A given microorganism can downregulate one or more complement pathways simultaneously, and the most common strategies found in bacteria, viruses, fungi, and parasites include the inactivation of complement proteins by secreted proteases, the production of surface proteins that mimic complement regulatory proteins, the acquisition of complement regulators from the host, the inhibition of C3 and C5 convertase activity or MAC formation, and the inactivation of antibodies through degradation or binding to their Fc portions (3).

Since *E. coli* has been shown to modulate key molecules to circumvent complement attack, we aim here to provide a comprehensive review of the interaction of this pathogen with the complement system by presenting the different mechanisms used by pathogenic *E. coli* to resist complement-mediated bacteriolysis.

## Complement Evasion Strategies by *E. coli*

### Presence of Capsule

Capsular polysaccharides are essential for bacterial virulence since they are involved in important biological processes including adhesion and resistance to host’s immune responses. Resistance to complement-mediated killing and phagocytosis are among the roles played by those structures (4).

The correlation between serum resistance and the presence of capsular polysaccharide in *E. coli* dates back to the 1970s and 1980s (5–11). Previous studies have shown that the K1 capsular polysaccharide is crucial for *E. coli* survival in the blood. K1 capsule production predominates among extraintestinal pathogenic *Escherichia coli* (ExPEC) strains. K1 *E. coli* strains may cause high levels of bacteremia, leading to meningeal invasion, what can be attributed to their capacity to evade host defense mechanisms (6, 12). Mutants deficient in K1 are unable to cause high-grade bacteremia and reach the blood–brain barrier in a neonatal rat model (12).

The K1 capsular polysaccharide of *E. coli* is composed of sialic acid. It is well known that FH, the main soluble regulator of the alternative pathway, binds polyanions such as sialic acid (13). As a consequence, downregulation of the alternative pathway occurs and may protect encapsulated bacteria from complement attack.

Interestingly, it has been demonstrated by Vermeulen and colleagues that there is a threshold level of K1 capsular content needed to afford protection against lysis promoted by the serum. Increased production of K1 capsule correlates with virulence by reducing complement activation and opsonization of *E. coli* (14). Further studies by Leying et al. (15) confirmed and extended previous findings showing that K1 expression is a prerequisite for serum resistance, since loss of capacity to produce K1 leads to serum susceptibility.

More recently, the K2 capsule, present in ExPEC, was also shown to play a significant role in pathogenesis. The K2 capsule-deficient mutant is more susceptible to the bactericidal activity of the serum. Complementation of the mutant with the genes coding for the K2 capsule restored the wild-type phenotype, enhancing serum resistance and bacterial survival in urine and kidneys (16). A protective role for the K54 capsule of an ExPEC strain against complement activation *via* the alternative pathway was also reported by Russo and colleagues (17).

### Secretion of Proteases That Directly Inactivate Complement

In *E. coli*, two autotransporters with serine protease activity have been described to inactivate complement components: extracellular serine protease P (EspP) and protein involved in colonization (Pic). Both proteases are secreted by enterohemorrhagic *E. coli* (EHEC) (18). Pic is also produced by enteroaggregative *E. coli* (EAEC) (19), uropathogenic *E. coli* (UPEC) (20), and enteropathogenic *E. coli* (21).

Extracellular serine protease P and Pic target an array of host physiological substrates. EspP, a 105-kDa protease, was first described to cleave pepsin A and human coagulation factor V, thus contributing to hemorrhagic colitis (22). Later, EspP was shown to inactivate complement proteins. Both purified and serum C3/C3b and C5 are cleaved by EspP, but the protease does not target FH or FI, indicating that this bacterial enzyme is not that promiscuous (23). Moreover, intact FH and FI can be helpful in aiding bacteria to escape complement activation by the alternative pathway. Reduced complement activity was observed in the presence of EspP and could be attributed to cleavage of C5 by this protease. Normal levels of complement activation were detected with the inactive EspP S263A mutant (23).

An even greater number of biological functions have been described for Pic. This 116-kDa secreted autotransporter protein confers serum resistance, possesses hemagglutinin and mucinolytic activities *in vitro*, cleaves surface glycoproteins involved in leukocyte trafficking, migration, and inflammation, and, similar to EspP, also cleaves coagulation factor V (19–21, 24–26). The first work to assess the role of Pic in *E. coli* serum resistance was published in 1999 by Henderson and colleagues (19). This group clearly demonstrated that Pic could protect *E. coli* DH5α from complement-mediated bacteriolysis. However, the mechanisms underlying serum resistance conferred by Pic were investigated only recently. Similar to EspP, Pic was shown to cleave complement proteins from all three pathways including C2, C3, C3b, C4, and C4b. Other molecules such as C1q, C5, and FH were not degraded (27). As expected, pretreatment of normal human serum with Pic decreased complement activation mediated by the classic, the lectin, and the alternative pathways. Interestingly, C3 cleavage by Pic produces a C3b-like molecule that is further degraded by FI/FH, thus indicating that Pic works synergistically with host complement molecules to inactivate C3 (27). It is worth to mention that strains of UPEC and EAEC known to secrete Pic may eventually reach the bloodstream, and cause bacteremia and sepsis (28, 29). Moreover, antibodies to Pic are produced during natural infection being detected in serum samples of children with acute diarrhea caused by EAEC (30).

*Escherichia coli* O157:H7, one of the Shiga toxin-producing *E. coli*, also secretes StcE (secreted protease of C1 esterase inhibitor from EHEC), a metalloprotease that specifically cleaves both purified and serum C1-INH, a regulator of the classical pathway of complement. StcE activity is specific for C1-INH, since no other serum proteins or extracellular matrix components are hydrolyzed by this metalloprotease (31). The functional consequences of StcE–C1-INH interaction were further assessed, indicating that StcE interferes with the complement cascade in a very particular way. StcE inhibits the classical complement-mediated erythrocyte lysis by potentiating the action of C1-INH. It has been demonstrated that the bacterial protease guides C1-INH to the surface of erythrocytes, locally downregulating complement activation. StcE binds to the aminoterminal portion of C1-INH, and cleavage sites have been localized to this heavily glycosylated domain. Such interaction does not affect the binding of C1-INH C-terminal serpin domain to target molecules or cells. Furthermore, this secreted metalloprotease was shown to enhance survival of a serum-sensitive *E. coli* K12 strain. It is assumed that by sequestering C1-INH to the surface of target cells, StcE may aid both the bacterium and the host to escape the harmful consequences of complement activation (31). Given that intestinal colonization by EHEC does not lead to a systemic infection, one may wonder how complement modulation by StcE would contribute to bacterial pathogenesis. It is presumed that by binding to host cells, StcE may be transported to sites distal to *E. coli* infection in the same way as the Shiga toxin, thus regulating C1-INH functions far beyond the original site of bacterial colonization. In addition, the recruitment of C1-INH to cells may also benefit *E. coli* O157:H7 by limiting their opsonization by C3b as well as the production of the inflammatory mediators C3a and C5a (31).

In addition to the secreted proteases mentioned above, *E. coli* produces a periplasmic protease named Prc or Tsp (Tail-specific protease). Prc exerts multiple functions and was first described as being involved in the C-terminal processing of penicillin-binding protein 3 (32). More recently, this protease was shown to contribute to *E. coli* complement evasion by interfering with the classical pathway activation (33). A *prc* mutant generated from a pathogenic *E. coli* K1 strain failed to induce a high degree of bacteremia, and presented increased levels of C3b and MAC deposition. Prc lacks proteolytic activity against complement proteins, but its protease function, namely the degradation of proteins with non-polar C termini (34), is critical for *E. coli* survival in serum (33). As the deletion of *prc* alters the outer membrane protein profile of *E. coli*, it has been suggested that *prc* ablation enhances outer membrane permeability, thus rendering bacteria more prone to MAC deposition (33).

The production of proteases is certainly an important feature equipping a number of pathogens with the ability to survive in the host, thus favoring their invasive potential and increasing disease severity.

### Complement Inactivation by Host-Acquired Complement Proteins

The complement system is tightly regulated by negative regulatory proteins to protect cells from the inflammatory and lytic effects resulting from complement activation. A number of pathogens recruit those complement regulators in order to survive and disseminate in the host. Acquisition of host complement soluble regulatory proteins is one of the strategies adopted by serum-resistant *E. coli* strains. C4BP and FH, the soluble regulators of the classical/lectin and alternative pathways, respectively, are among the complement molecules recruited by those strains.

The outer membrane protein A (OmpA) is an abundant and highly conserved protein of *E. coli*, and has been shown to contribute to serum resistance of neonatal meningitis *E. coli* (NMEC) both *in vitro* and *in vivo*. In 1991, Weiser and Gotschlich demonstrated that *E. coli* K1 deficient in OmpA is more susceptible to complement-mediated lysis by the classical pathway (35). Ten years later, Prasadarao et al. reported the interaction of OmpA with C4BP, what could potentially allow complement downregulation on the bacterial surface, contributing to survival and dissemination (36). The OmpA–C4BP interaction was then characterized in detail. The aminoterminal portion of OmpA binds to the complement control protein domain 3 of C4BP α-chain, and this interaction could be inhibited by synthetic peptides. In addition, blocking C4BP binding to OmpA rendered bacteria more sensitive to lysis by normal human serum (36). Further studies by the same group demonstrated that postexponential phase OmpA + *E. coli* K1 circumvent serum bactericidal activity less efficiently than log phase bacteria because they recruit less C4BP to their surface. As a consequence, the deposition of C3b in log phase bacteria is lower, allowing them to avoid MAC-mediated killing more efficiently. C4BP bound to OmpA + *E. coli* K1 remains functional, acting as cofactor for FI-mediated inactivation of both C3b and C4b (37).

Besides OmpA, another outer membrane protein of approximately 34 kDa called new lipoprotein I (NlpI) (38) facilitates deposition of the complement regulator C4BP on the surface of NMEC to avoid complement activation by the classical pathway (39). Upon incubation with normal human serum, C3b and MAC deposition on *E. coli* mutant for the *nlpI* gene was more pronounced than that observed on wild-type bacteria. The *nlpI* mutant and the wild-type *E. coli* strains show similar C1q deposition, but the mutant strain binds less C4BP. In addition, wild-type bacteria survival is impaired in C4BP-depleted serum, indicating that the recruitment of this complement regulatory protein contributes to serum resistance. Nevertheless, heat-inactivated NlpI antiserum could not block the recruitment of C4BP by wild-type bacteria, suggesting that NlpI may not interact directly with C4BP. Assays involving single and double knockouts for OmpA and NlpI suggest that both proteins work coordinately to recruit C4BP to the surface of NMEC, but further assays are necessary to clarify this issue (39).

More recently, outer membrane protein W (OmpW), a protein present in a large number of Gram-negative bacteria, was shown to bind FH, the main soluble regulator of the alternative pathway of complement (40). OmpW is involved in diverse biological processes including the response against environmental stress (41), resistance to antibiotics (42–44), and has been reported to contribute to bacterial virulence (45, 46). Li and colleagues have demonstrated that OmpW is upregulated when bacteria are incubated in the presence of normal human serum (40). Based on these findings, they wondered if OmpW could contribute to serum resistance. Serum bactericidal assays were performed showing that OmpW confers a certain degree of protection against complement-mediated killing. A mutant strain lacking the *ompW* gene presented lower survival in EGTA-Mg^2+^ supplemented serum compared to the wild-type and the *ompW* complemented strains, suggesting that OmpW confers protection against the alternative pathway of complement. Additional assays will be required to investigate whether FH bound to OmpW remains functional.

Infections caused by EHEC are an important cause of hemolytic uremic syndrome (HUS). These bacteria produce Shiga toxins (Stxs), and Stx2 represents a major virulence factor of EHEC, contributing to HUS pathogenesis. It has been demonstrated that Stx2 binds FH and other members of the FH family such as FHL-1 and FH-related protein-1 (FHR-1) (47, 48). While Stx2-bound FH/FHL-1 retains cofactor activity in the fluid phase, the interaction of Stx2 with FH on the cell surface compromises its cofactor function. FH binds to cell surfaces through short consensus repeats 18–20, which represent one of the Stx2-binding sites on the FH molecule. As a consequence, FH regulatory function would be impaired on the cell surface, leading to tissue damage. It is hypothesized that complement activation mediated by Stxs may contribute to the severe kidney injury observed during the course of HUS (47). Of note, it has also been shown that both FHL-1 and FHR-1 compete with FH for binding to Stx2. Competition assays performed with physiological molar ratios of complement regulatory proteins resulted in a reduced cofactor activity when FHR-1 and FH were used. Obviously, the *in vivo* scenario must be far more complex than the picture drawn from *in vitro* data, but these studies pave the way for understanding the role of complement in the pathogenesis of HUS caused by EHEC.

In summary, the interaction of *E. coli* with complement regulatory proteins contribute to pathogenesis either by inactivating the complement cascade and enhancing bacterial survival or by potentiating complement activation, ultimately causing tissue damage.

## Concluding Remarks

Pathogens use a range of strategies that allow them to survive and disseminate in the host. Avoiding complement attack is a prerequisite to successfully persist in the colonized organism, and extraintestinal pathogenic *E. coli* have evolved diverse strategies to resist complement-mediated killing. Given that high levels of bacteremia are required for certain *E. coli* to cross the blood–brain barrier, notably those that cause meningitis in neonates, it is clear that these pathogenic strains are equipped with sophisticated mechanisms to efficiently overcome the deleterious effects of complement activation.

**Figure 1:**
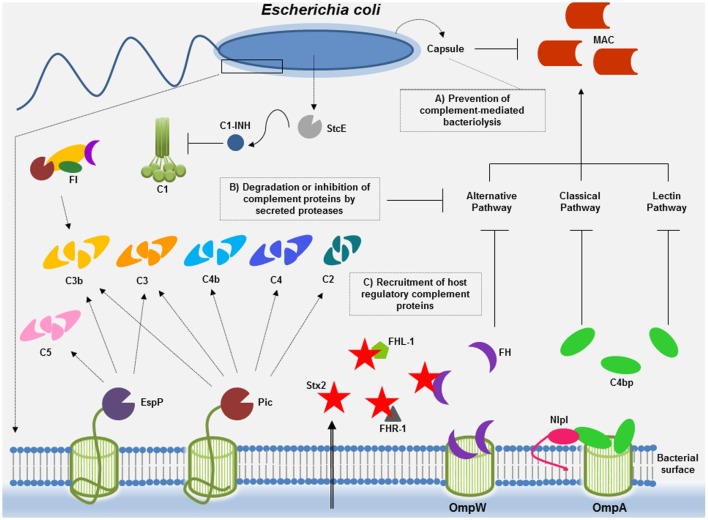
**Complement evasion strategies of *Escherichia coli***. To overcome complement attack, *E. coli* has evolved diverse immune evasion strategies: **(A)** capsular polysaccharide may confer resistance to complement-mediated killing. Factor H (FH), the main soluble regulator of the alternative pathway, may interact with polyanions such as sialic acid, present in the K1 capsular polysaccharide of *E. coli*. Downregulation of the alternative pathway may occur, thereby impairing membrane attack complex (MAC) deposition. **(B)** *E. coli* proteases such as Pic and extracellular serine protease P (EspP) may cleave complement components of all three pathways or, in the case of StcE, may recruit C1 inhibitor (C1-INH) to the surface of target cells, locally downregulating complement activation by the classical pathway. **(C)** Acquisition of the soluble complement regulators FH and C4b-binding protein (C4BP) by outer membrane protein W (OmpW) and by outer membrane protein A (OmpA)/new lipoprotein I (NlpI) may allow downregulation of the alternative and the classical/lectin pathways of complement, respectively. Binding of proteins of the FH family to Stx2 may result in complement activation contributing to tissue damage (renal injury) observed during the course of hemolytic uremic syndrome caused by enterohemorrhagic *E. coli*.

In *E. coli*, mechanisms conferring serum resistance include the protection afforded by capsular polysaccharide, the degradation of complement proteins into non-functional fragments by secreted proteases, and the recruitment of host negative complement regulatory proteins (Figure 1). Unraveling the mechanisms beyond complement inactivation by *E. coli* may not only contribute to our understanding of the bacterial pathogenesis but also pave the way for the development of new therapeutics targeting microbial immune evasion strategies.

## Author Contributions

AA participated in the drafting of the article and prepared the figure; AB participated in the drafting of the article and performed a critical revision of the final version.

## Conflict of Interest Statement

The authors do not have a commercial or other association that might pose a conflict of interest.
